# Editorial: Psychology and mathematics education

**DOI:** 10.3389/fpsyg.2023.1243419

**Published:** 2023-07-27

**Authors:** Laura Martignon, Karin Binder, Katharina Loibl, Laura Macchi, Gila Hanna

**Affiliations:** ^1^Institute of Mathematics and Computing, Ludwigsburg University of Education, Ludwigsburg, Germany; ^2^Institute of Mathematics, Ludwig Maximilian University, Munich, Germany; ^3^Institute of Psychology, University of Education Freiburg, Freiburg, Germany; ^4^Department of Psychology, University of Milano Bicocca, Milano, Italy; ^5^Department of Curriculum, Teaching and Learning at the University of Toronto, Toronto, ON, Canada

**Keywords:** mathematics, psychology, pedagogy, classroom, learning

We use numbers and fractions every day, for example when we are doing our shopping or baking a cake. But mathematics is, of course, much more: it is the language of science, or, to use Galileo's words, “the book of Nature is written in mathematical language” (Galileo, 1623) and some mathematical competencies beyond basic arithmetic are required in most professions. Basic mathematics, i.e., elementary arithmetic, elementary geometry and some elements of calculus, is taught in school, not just for everyday life, but as a tool for many different professions. In school, however, mathematics is either “loved” or “hated”, as Hersh and John-Steiner masterfully describe in their book “Loving and Hating Mathematics” (Hersh and John-Steiner, 2010). Research in mathematics education has definitely contributed to reducing school students' hatred of mathematics and this reduction may be seen as one of its many goals.

In contrast with mathematics, the field of mathematics education is strongly interdisciplinary; the closest field to influence it directly is psychology. In fact, mathematics education is consistently shaped by both behavioral and cognitive perspectives, since so many factors—the power of visualizations, the effect of representation formats, but also factors like gender, self-efficacy, etc.—influence and sometimes determine students' performance.

Our aim for this Research Topic and for the collection of papers we are now publishing has thus been to illustrate the relevance of such various psychological perspectives for mathematics education using the contributions of colleagues from around the world. All the contributions we have collected address these interdisciplinary perspectives explicitly or implicitly.

We were surprised by the success of our Research Topic, which was perhaps triggered by the wide range of possible research directions addressed by its general title. The largest section of the papers presents empirical, original research carried out by a wide variety of specialists, with interesting results and suggestions for educators in the classroom. Other papers, which describe psychological features of mathematics education, review factual evidence and the relevant literature. Thus, the collection we present has a descriptive as well as a pragmatic and a prescriptive orientation. It consists of 39 contributions by 109 authors, including 29 original research articles, four brief research reports, three reviews, and three conceptual analyses.

Due to the large number of original research articles, we have decided to arrange them according to different sub-topics, which we list here:

1. Visualizations and representation formats
2. Reasoning, argumentation and biases in connection with mathematics
3. The influence of motor skills
4. Mathematics anxiety as a determining factor
5. Gender and its consequences for students' performance
6. Cultural differences, self-efficacy and consequences for mathematical development
7. Teachers' views, beliefs and culture in connection with teaching mathematics.

We now proceed to describe these sub-topics and cite examples from the corresponding papers.

## 1. Visualizations and representation formats—their fostering of mathematical intuitions

As we wrote in the overview for this Research Topic, a great inspiration for our endeavor was provided by the work of Herbert Simon with his concept of bounded rationality and its direct descendent, ecological rationality. The ecological rationality perspective allows a novel way of viewing typical aspects of mathematics education, pointing at its fundamental links with cognitive psychology.

We cite here one of Herbert Simon's most famous statements concerning the fundamental importance of representation for solving problems:

*“Solving a problem simply means representing it so as to make the solution transparent.” (Simon*, *1996**, p. 132)*

According to Simon, a solution becomes transparent if it emerges from the representation of the structure in terms of which the problem has been modeled. The structure results from an effort of adaptation between the endeavors to solve it and the conceptual constructions of the mind. Adopting the phrasing of Gigerenzer and his school, an adaptation is successful if it is ecologically rational (c.f. Gigerenzer et al., 1999). Ecological rationality is thus considered a fundamental characteristic of successful representations: it refers to behaviors and thought processes that are adaptive and goal-oriented in the context of the *representational environment* in which the organism is situated.

Constructing a representation that makes a problem easily solvable provides evolutionary advantages in terms of time resources to agents adopting it (Martignon et al., 2020). The most popular example from mathematics is the representation of numbers based on the decimal number system. This example is so fundamental for Mathematics in every-day life, that it deserves a short digression: the positional representation, as is well known, made its way into the western world several centuries after its inception in India. Dysfunctional systems like Roman numerals had forced people in Europe to outsource their counting and computing: during the late Middle Ages and early Renaissance the Abakists did the numerical operations for businessmen, translating Roman numerals into strings of marbles and working with them on their abaci. Their results were then translated back into Roman numerals. Herbert Simon had wondered about the “theoretical” reasons that make the Hindu-Arabic number system so much more adaptive to our minds than Roman numerals. In fact, he wrote:

*“We all believe that arithmetic has become easier since Arabic numerals and place notation replaced Roman numerals, although I know of no theoretical treatment that explains why” (Simon*, *1996**)*.

**Figure 1:**
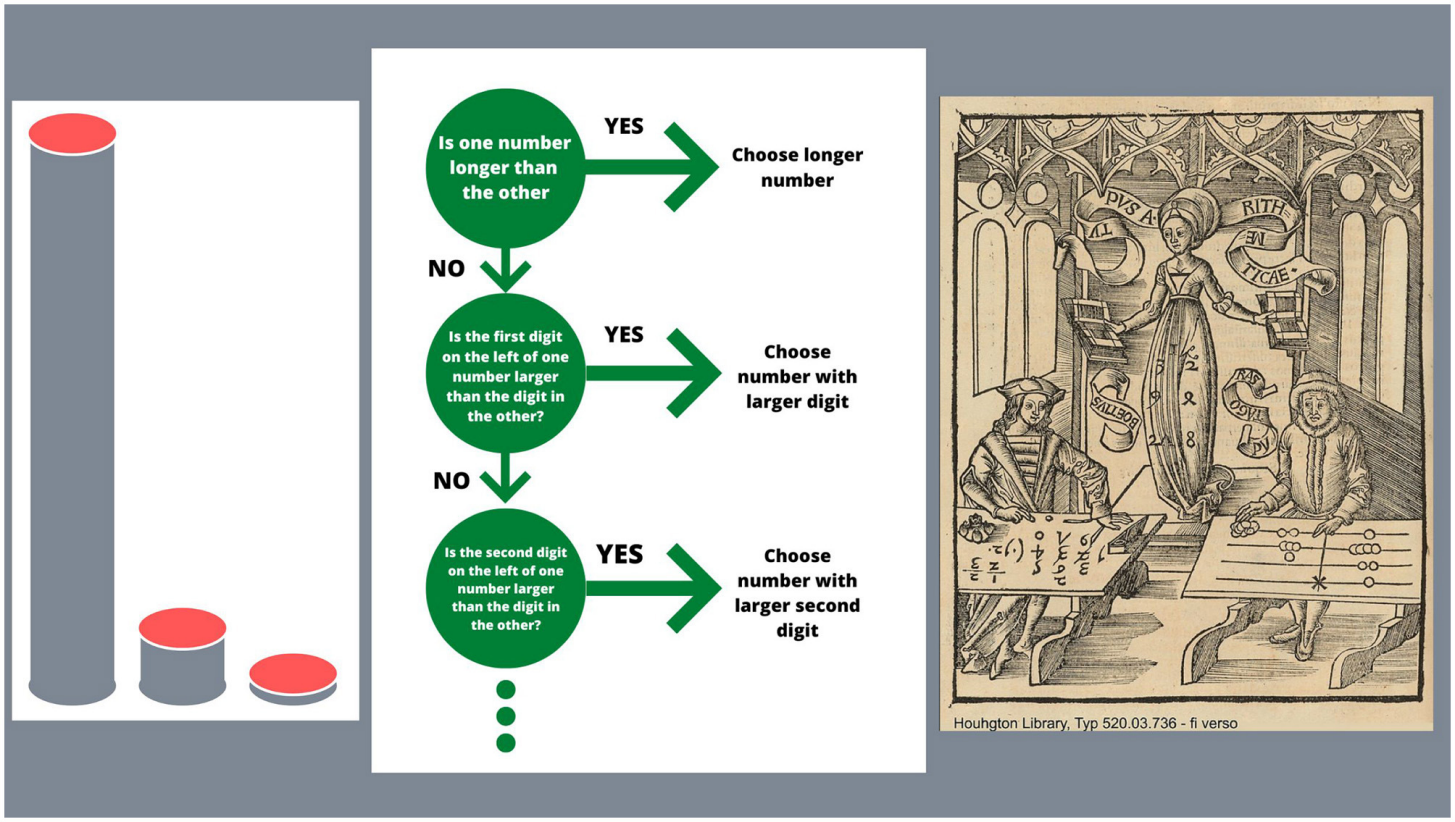
Aspects of the decimal representation system. On the left side, three powers of 10 are illustrated by means of cylinders of corresponding heights. They correspond to the weights of the simple non-compensatory linear model, which characterizes our number system. In the center of the figure, a simple, fast, and frugal tree describes the quick yet sequential decision procedure we use for number comparison. On the right side, the reader sees a famous illustration of Arithmetic represented through an allegory by Reisch in the Margarita Philosophica, who contemplates with a benevolent smile the “algarist” (image source: Typ 520.03.736, Houghton Library, Harvard University). The algarist performs computations based on the positional system, while the “abakist”, on the other side of the illustration, uses marbles for computation with his abakus. The work of abakists was fundamental during the beginnings of the Renaissance, when businessmen employed them to perform all their computations. The decimal positional system made arithmetic calculations simple, transparent, and thus accessible for everyone.

The studies of ecological rationality and simple heuristics by Gigerenzer and his school have provided good explanations (see Martignon et al., 2020 for a brief synthesis of the main arguments): the mind/brain, as has been empirically demonstrated, is akin to a sequential, lexicographic treatment of features for comparison tasks or classifications. In the case of two numbers in the decimal positional system their coefficients of powers of 10 are the features a comparison is based on. In fact, when having to compare, say 3.456 and 3.461, for instance, we first check that they are equally long. i.e., they have the same number of digits. Then we start checing each digit from left to right, stopping when we find a difference in digits and determine the largest number accordingly. This procedure can be described by a fast-and-fugal tree, as in the illustration at the center of Figure 1. A fast-and-frugal tree is a simple lexicographic decision tool that proceeds in a sequential way. Its success is the consequence of the non-compensatory weights of its features, as, in the case of numbers, powers of 10 (Figure 1, Left).

Obviously, the decimal positional system has other fundamental advantages for computing.

The illustration in the right panel of Figure 1 represents an illustration from the Margarita Philosophica (by Gregor Reisch) of 1503. Here the allegory of Arithmetica in the center, looks benevolently toward the “Algarist”, on the left side, who works with numbers in the positional system. On the right side, the Abakist, computes with marbles.

The relevance of ecological rationality for our Research Topic is made evident in several of the papers here. In fact, several authors address aspects of visualizations and representations in mathematical contexts which foster the adaptation of the mind to the mathematical contexts involved. Mathematics education treats representations of mathematical situations and entities as a fundamental aspect of didactics in the classroom. Of course, multiple representations of mathematical entities are possible: the advantages of juggling between them have been treated extensively by Dreher et al. (2016). However, as work in this Research Topic demonstrates, for many types of problem, such as fractions and probabilities, different representations can be mathematically equivalent, yet be far from cognitively equivalent. That is, some representations are more adaptive and advantageous than others because they are aligned to the cognitive systems of human problem-solvers. Representing numbers geometrically has an ecological aspect, and the paper by Kempen et al. demonstrates empirically how figurative numbers enhance numerical understanding.

Approaching the realm of functions in mathematics, for instance, we recall that it would be cumbersome to think about them without the coordinate system introduced in the early seventeenth century by Descartes. Today, much progress has been achieved in dynamic visualizations of functions in coordinate systems. These visualizations allow us to go even further and to greatly improve the understanding of certain aspects of functions. In this connection, we mention the paper by Rolfes et al. in which the authors show that students can learn covariational aspects of the concept of function significantly better with dynamic visualizations than with static representations. However, as they also specify, there seems to be no significant difference in learning with linear or interactive dynamic visualizations.

A natural question related to representations seems to be whether self-generated drawings can always be of help. The answer seems to be that this is not always the case. Self-generated drawings are not always adaptive. In fact, only aspects of them, like their quality, may be of value. Krawitz et al. carried out a replication and elaboration study on the negative effect of self-generated drawings on the number of students' linear overgeneralisation and problem-solving performance. The drawing quality, but the not visual monitoring, affected the number of over generalizations. These results indicate the great importance of the quality of drawing as a strategy for problem-solving.

One mathematical field that has profited immensely from the search for adaptive representations is probability and statistics. Statistical situations concerning data sets profit hugely from dynamic representations. Hood et al. found that dynamic graphs in digital publications can potentially be used for communicating interactions (and other complex relationships) effectively. They are not, however, a panacea for people's challenges in understanding complicated data and more work is needed to take effective advantage of opportunities in digital data presentations.

Probabilistic inference has also profited greatly from adaptive representations: we recall here that it has been a milestone in the field of probabilistic reasoning to discover how certain representations and visualizations foster Bayesian reasoning (Gigerenzer and Hoffrage, 1995) while others hamper it.

The paper by Eichler et al. is especially inspiring, quite in the spirit of ecological rationality, because of the empirically supported claim that people's strategies for solving Bayesian tasks are *triggered* by corresponding representations, exactly in the sense of Herbert Simon.

Another relevant finding in this field is that the characteristic of visualization making the nested-sets structure of a Bayesian situation transparent has a facilitating effect on people's Bayesian reasoning (Macchi, 2003). Trees and double trees with nodes representing natural frequencies have been proven very effective in this context. A practical extension of the tree format with natural frequencies is the so-called “frequency net” proposed by Binder et al.. Here the disposition allows for sequential treatment in four directions but also provides a view of all relevant frequencies of conjunctions at a glance.

Another question that arises in the context of natural frequencies is whether younger children may exhibit forms of Bayesian reasoning when presented with simple formats of information. The paper by Till et al. presents an intervention study showing that primary school children at the age of nine to ten can understand probabilities and solve Bayesian problems using natural frequency formats. Hence, natural frequencies appear to be a suitable representation for grasping probabilities at an early stage and thus might support understanding more abstract contexts in higher grades.

It is a crucial result, that people may perform correct Bayesian inferences without recurring at all to numerical formats of information: Leuders et al. analyzed how people update their hypotheses based on uncertain evidence (e.g., teachers' updating their assumptions based on students' solutions), when they only have access to non-numerical information. They showed that people need strong support to apply a rational Bayesian strategy and otherwise resort to biased strategies for processing information–analogous to the strategies found in numerical settings.

The review by Neth et al. solves several well-known problems by representing them more transparently. Politely phrased as re-framing representational effects and suggesting a change in perspective, a more apt description of their achievement is to effectively put an end to academic debates and scientific practices that are sustained by obscure abstractions and idiosyncratic terminologies. Encouraged by editors and reviewers, the authors resisted the temptation to distribute their insights across several articles. The published product is long and detailed, but rewards its readers by seeing *how* numerous scientific puzzles and their solutions are alternative perspectives on a shared representational construct.

## 2. Reasoning, argumentation, and biases in connection with mathematics

Reasoning is the basis not only of mathematical thought, but of critical thinking in general. In an era of fake news and propaganda proliferated by social media, critical thinking acquires even greater relevance and has been declared one of the competencies of the 21st century by the OECD.

The work by Macchi et al. is represented in this Research Topic by two papers related to the improvement of logical and mathematical performance (Bagassi et al.; Bagassi and Macchi) of children through a pragmatic approach, on the one hand, and on the possibility of facilitating problem-solving by viewing it as the overcoming of misunderstandings, on the other.

Another important field in the realm of reasoning deals with how humans cope with syllogisms. These used to be the basis of reasoning in the traditional approach to rigorous thinking. Syllogisms and how humans handle them have been a matter of research through the centuries. They are the essence of classical logic. But in heuristic decision-making, which definitely takes place when students approach problem-solving in mathematics, less “classical” logics may play an important role. In Chapter 3 of “The Science of the Artificial” Simon recommends the use of multiple logics and in Chapter 5, he explains how

*“[m]ultiple logics may become necessary when approaching heuristic decision making” (Simon*, *1996**)*.

The paper by Vargas et al. introduces variations on syllogistic experimental tasks by (1) reshaping the pragmatics of the communication situations faced along the dimension of cooperative vs. adversarial attitudes and (2) rendering explicit the construction of counter-examples. It presents evidence on a significant switch in participants' performance and the strategies they employ while reasoning.

The question on how to foster argumentation skills deals with the design of adequate learning environments and can be influenced, as Sommerhoff et al., show, by whether a sequential or a concurrent instructional approach is used in the classroom. Their paper highlights that sequential and concurrent approaches are both effective in supporting the resources underlying mathematical argumentation and proof skills; however, the concurrent approach can have slightly better effects on mathematical argumentation skills, especially in the case of weaker students.

The paper with most views so far is the one by Bruckmaier et al. on the cognitive illusions studied by Kahneman and Tversky, which released a flurry of fundamental investigations on human reasoning. They provide a unified framework for the basic treatment of the classical teasers analyzed by the school associated with Tversky and Kahneman.

Playing games may sharpen reasoning and lead to concept formation. Özel et al. report having worked with children from 8 to 10 years old, who played different versions of a code-breaking game in guided game-based instruction. After this process, a post-test showed that children were remarkably sensitive to key principles in their mathematical reasoning when dealing with information. This adds to evidence that game-based instruction can be a powerful tool for making mathematics moreintuitive.

Through playing games the phenomenon of “help-seeking”, which is so relevant in the context of learning and problem-solving, can be efficiently analyzed, as Taylor et al.. They show that help-seeking is not correlated with a real need for help. The important paper by Jonsson et al. is devoted to how mathematical understanding can be fostered by creativity and cognitive proficiency.

Pursuing another line, in their paper, Reinhold et al. empirically analyzed the biases—the natural number bias in particular—that make working with fractions difficult, especially for low-achieving students.

The paper by Yang et al. analyzes how concept formation, and understanding of categories fosters analytical and mathematical competencies.

## 3. The influence of individual learner characteristics

### 3.1. The influence of motor skills

The paper by Fischer et al. on the surprising effect of fine motor skills to mathematical insight is particularly relevant. This is the only paper in the whole Research Topic collection that treats connections between movement and mathematical competency.

### 3.2. Mathematics anxiety as a determining factor

Three papers in our collection treat mathematics anxiety in connection with mathematics in the context of school (Maldonado Moscoso et al.; Primi et al.) Among the psychological factors that trigger impairments in mathematics, mathematics anxiety has been suggested to play a key role. It has been defined as feelings of apprehension and increased physiological reactivity when individuals have to manipulate numbers, solve mathematical problems or when they are exposed to an evaluative situation connected with mathematics. Mathematics anxiety involves psychological arousal, negative cognitions, escape and/or avoidance behaviors and, when the individual cannot avoid the situation, performance deficits. It is described as a multidimensional construct that is related to, but distinct from, other forms of anxiety, such as trait, social or test anxiety. Mathematics anxiety has been shown to hinder mathematics performance. This phenomenon is very common not just among school students. Adults suffer from it as well.

The paper by Primi et al. describes a new scale for measuring it already in young children.

The paper by Moliner et al. describes a how peer tutoring among school students can become a factor thast reduces math anxiety.

### 3.3. Gender and its consequences on students' performance

In the paper by Uclés et al. on “*Gender Differences in Visuospatial Abilities and Complex Mathematical Problem Solving*” the authors provide empirical evidence that students with the ability to solve complex mathematical problems exhibit stronger spatial skills. It also shows that boys and girls present similar spatial abilities, and that there is no significant interaction between the ability to solve complex problems and gender.

### 3.4. Cultural differences, self-efficacy, and consequences for mathematical development

The role of self-efficacy for mathematical development has become ever more evident since the discoveries of Bandura in the second half of the 20th century (Bandura, 1997). The paper by Siefer et al. reveals that including written data (notes) and non-verbal data (gestures and actions) leads to a more accurate analysis of self-explanations than an analysis solely based on verbal data. This influence is even stronger for the categorization of self-explanations as “adequate” or “inadequate”.

In the paper by Siefer et al. the authors explore the potential multi-dimensionality of self-efficacy focused on three task characteristics:

1. the representational format,
2. embedding in a real-life context,
3. the required operation.

The paper highlights the fact that even within a specific content domain students' self-efficacy can and should be considered a multi-dimensional construct.

The paper by Salle describes how self-explanation, gestures and notes trigger self-assurance and self-efficacy.

The paper by Zakariya addresses causal relationships between the previous and current mathematics performance of undergraduate students.

Pursuing another line, in their paper Meng et al. deal with the thought-provoking topic of the influence of specific cultural phenomena in connection with self-efficacy. In fact, it treats cultural aspects which are apparently more specific to upbringing in China, and shows that these aspects have an influence on factors analogous to self-efficacy when dealing with mathematical tasks.

Findings by Wang and Sperling revealed that those interventions grounded in metacognition-oriented theories and those interventions that targeted multiple strategies including cognitive, metacognitive, and motivational, tended to yield effective increases in both mathematics achievement and self-regulated learning.

## 4. Teachers' views, beliefs, and culture in connection with teaching mathematics

The role of teachers, their training and their views in the discussion on mathematics education is treated by two papers in the collection.

The paper by Patterson et al. shows the positive effects of special units of teacher training on the performance of students. The findings indicate that students profit from their teacher's participation in special training interventions.

The paper by Tanas et al. addresses how the views of teachers on technology and their perceived ease of technology use affects their use of technology in the mathematics classroom. These new tools provide education with many new opportunities, but their application often meets with a variety of difficulties. Many of those difficulties are general and appear across different areas of technology use. The paper confirms that perceived usefulness has a stronger direct impact on technology use and that user friendly technology increases use.

Besides research articles, our Research Topic contains two research reports, one systematic review, three conceptual analyses, two review articles and ends with an opinion.

## 5. Further contributions: research reports, systematic review, conceptual analyses, review article and opinion

### 5.1. Research reports

Sturm et al. report on an empirical study on how the attitudes and beliefs of young students correlate with their problem-solving performance. They also claim that this correlation can be affected by student participation in a training programme.

In his brief research report Rolfes treats the interpretations of pictorial charts involving differences in areas and differences in volumes, as understood by readers of popular reports. His claim is that readers do not seem to interpret two-dimensional pictures of three-dimensional objects spatially.

### 5.2. Systematic review

The paper Wang et al. analyzes findings on social-cognitive self-regulated learning and discuss implications for good practices in the classroom.

### 5.3. Conceptual analyses

In Bertram the author examines future directions in research on digital games in mathematics and computer science education. She highlights the importance of a sound psychological foundation for the development of learning games and the need for interdisciplinary research projects and randomized controlled experimental designs to evaluate the effectiveness of games and game features.

The analysis of Kramer on iconic mathematics is extremely pertinent to the realm of ecological rationality in the context of mathematics education. It reminds us of the necessity of reviewing representations of mathematical entities and processes that produce features that are appealing to the mind/body and thus become easy to grasp.

The analysis by Kurdoglu et al. describes one step in the conceptual view on uncertainty and is therefore relevant for the realm of decision-making, having connotations (implications?) that are meaningful for the teaching of probability. It deals with complete uncertainty, a situation that goes beyond mathematically structured scenarios. Under very high levels of uncertainty, decision-makers rely on heuristics to no avail. Kurdoglu et al. posit that eristic reasoning (i.e. self-serving inferences for hedonic pursuits), rather than heuristic reasoning, is adaptive when uncertainty is extreme. They explain how decision-makers can benefit from heuristic vs. eristic reasoning under different levels of uncertainty. Although the authors establish no immediate connection with mathematics in the classroom, their approach is novel and clearly relevant.

### 5.4. Review article

In Barrocas et al. the authors mostly review the large collection of their own discoveries concerning finger-counting as related to later arithmetic abilities. Their report fits in perfectly with the intention of the Research Topic.

### 5.5. Opinion

As a final perspective from the Research Topic, we cite here the paper by Simplicio et al. Here the authors insist that results from research should find their way into classrooms, but they see the need for more integration of different perspectives and fruitful collaborations between researchers of different disciplines and educators. Only then, they claim, are there real chances of transferring results from basic research into educational practice. Yet, they also point out that, as has been said by Minshall (2009), “knowledge transfer is a ‘contact sport'; it works best when people meet to exchange ideas, … and spot new opportunities”.

We definitely agree with their statements and conclude the description of our Research Topic with the hope that more steps toward the integration of research on the psychology and even on the neuroscience of mathematics acquisition are soon taken at all levels of research and implementation.

## Author contributions

LMar prepared the draft of the editorial, which was revised and completed by KB and KL. KB and KL had previously sorted all papers in the Research Topic and classified them in meaningful categories, as listed in the editorial. LMac and GH fully agreed with the editorial in its present form.
